# Can peripheral blood be used as surrogate in detecting epidermal growth factor receptor mutation status in advanced non-small cell lung cancer patients? A meta-analysis

**DOI:** 10.18632/oncotarget.20291

**Published:** 2017-08-16

**Authors:** Xiaowei Mao, Yujun Zhang, Fangfang Xie, Xiaoxuan Zheng, Jiayuan Sun

**Affiliations:** ^1^ Department of Endoscopy and Pulmonary Medicine, Shanghai Chest Hospital, Shanghai Jiao Tong University, Shanghai 200030, PR China

**Keywords:** EGFR, peripheral blood, advanced NSCLC, mutation, meta-analysis

## Abstract

**Background:**

Apply peripheral blood as a surrogate for detecting epidermal growth factor receptor mutation status in tumor, also called liquid biopsy, has been reported to be a feasible method in patients with advanced non-small lung cancer. But the diagnostic yield varies in different studies.

**Methods:**

A meta-analysis was carried out to evaluate the sensitivity and specificity of peripheral blood in detection epidermal growth factor receptor mutation status in advanced non-small lung cancer patients. Publications up to October 2016 were searched using PubMed, Embase and Web of Science databases. Sensitivity, specificity and other parameters were pooled using the bivariate mixed-effects regression model.

**Results:**

Fifteen studies meeting the inclusion criteria were included. The pooled sensitivity, specificity, positive likelihood ratio, negative likelihood ratio and diagnostic odds ratio were 0.69 (95% CI: 0.59~0.78), 0.97 (95% CI: 0.94~0.99), 23.1 (95% CI: 11.6~46.1), 0.32 (95% CI: 0.23~0.44), 73 (95% CI: 33~159), respectively. The summary receiver operating characteristic curve was 0.93 (95% CI: 0.91–0.95).

**Discussion:**

Detecting epidermal growth factor receptor mutation in peripheral blood is a reliable and non-invasive method in patients with advanced non-small lung cancer. More sensitive detection methods are required to increase the sensitivity of liquid biopsy of ctDNA.

## INTRODUCTION

Lung cancer is the leading cause of cancer-related death worldwide and non-small cell lung cancer (NSCLC) is the major type [1–2]. Since most patients with NSCLC are diagnosed at an advanced stage, when curative procedures are not available [3]. In addition, platinum-based doublet chemotherapy has reaches its plateau [4]. Several prospective clinical studies on EGFR inhibitors have demonstrated their efficacies and less toxicity in patients harboring epidermal growth factor receptor (EGFR) activating mutations [5–8]. Exon 19 deletion and substitution of L with R at position 858 in exon 21 have accounted for the majority of the EGFR mutations [9]. NCCN guidelines suggest to test for EGFR status in advanced NSCLC (aNSCLC) prior to commencing EGFR-TKIs as the first-line therapy [10].

Nowadays, tissue biopsies are still regarded as the golden standard for EGFR mutation examination. However, tissue biopsy has limited ability in reflecting tumor heterogeneity, owning to its spatial and temporal snapshot nature. Furthermore, tissue biopsy is also invasive and occasionally would result in complication. Tissue biopsy can't apply to a significant fraction of advanced patients with prior treatments [11]. Liquid biopsy, containing ctDNA released from apoptotic or necrotic tumor cells, can potentially reflect the genetic profile of tumors [12–14]. Numerous studies have shown that EGFR status can be detected using ctDNA. However, the sensitivity of using different assays assessing EGFR status from ctDNA varies significantly, ranging from 22% to 94% [15–16].

In this study, we reviewed 15 manuscripts to investigate whether the peripheral blood can be used as a reliable surrogate specimen for detecting EGFR mutation status in aNSCLC patients when the tumor tissue is unavailable or inadequate.

## RESULTS

### Study selection

**Figure 1:**
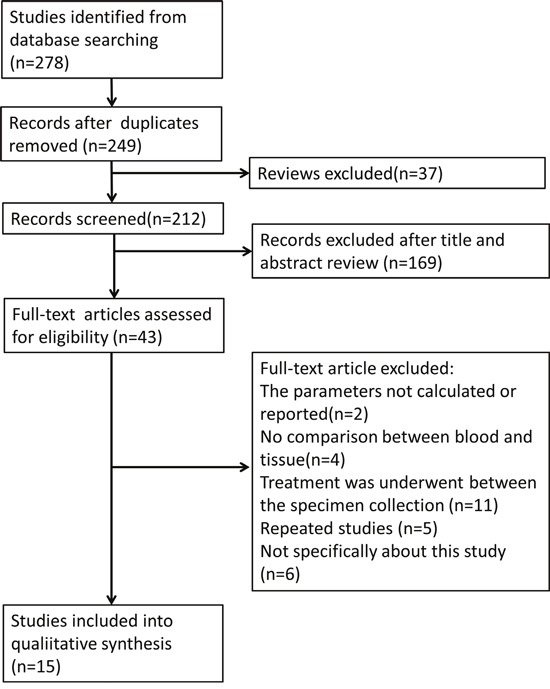
Flow chart for articles identified and included in this meta-analysis

Our search strategy identified 278 publications for consideration. Of these, 29 duplicated studies and 37 reviews were eliminated. Then, 169 were excluded based on review of the titles and abstracts. Of the 43 publications remained, full articles were obtained and reviewed, and another 28 studies were excluded for the following reasons: five were repeated articles; two had too little data to form the 2×2 table; four studies lacked the comparison of EFGR status between blood and tissue; eleven compared pre-treatment tissue and post-treatment blood; and six did not specifically pertain to the subject. (Figure 1) Fifteen publications meeting all of the inclusion criteria were considered for the meta-analysis.

### Study descriptions and quality assessment

The characteristics of the included studies are listed in Table 1. A total of 2094 patients were enrolled, ranging from 32 to 652 patients per study. Of all studies, six studies were prospective, two studies were retrospective, and the remaining seven studies did not specify the study type. Thirteen studies enrolled Asian patients and the other two only enrolled Caucasians. Twelve studies detected EGFR mutations using plasma, two studies used serum, and one study used ctDNA extracted from specimen combinated with plasma and serum. The amount of blood needed in eight studies is less than 6 ml, in 3 studies is more than 6 ml, and 4 studies didn't specify. The same detection method was applied in eleven studies and different detection methods were applied in the remaining four studies.

**Table 1:** Characteristics of eligible studies

First Author	Ethnicity	Design	Blood Sample	Blood Amount	Stage	Method (tissue)	Method (blood)	type	No. of Patients
Kimura et al. [17]	Asian	Retrospective	Serum	4ml	IIIB-IV	Direct sequencing	ARMS	ADC, SQCC, LCC	42
Bai et al. [18]	Asian	Prospective	Plasma	NA	IIIB-IV	DHPLC	DHPLC	ADC, SQCC, LCC	230
Yung et al. [16]	Asian	Prospective	Plasma	2-6	III-IV	dPCR	dPCR	NSCLC	32
Jiang et al. [19]	Asian	NA	Serum	5ml	IIIB-IV	ME-PCR	ME-PCR	ADC, SQCC, LCC	58
Xu et al. [20]	Asian	Retrospective	Plasma	NA	IIIB-IV	ARMS	ARMS	NSCLC	34
Zhang et al. [21]	Asian	NA	Plasma	5ml	IIIB-IV	mutant enriched liquidchip	mutant enriched liquidchip	NSCLC	86
Kim et al. [22]	Asian	Prospective	Plasma	6ml	IIIB-IV	Direct sequencing	PNA-PCR	ADC, SQCC	57
Wang et al. [16]	Asian	Prospective	Plasma	2ml	IIIB-IV	ARMS	ARMS	NSCLC	134
Li et al. [23]	Asian	NA	Plasma	4ml	IIIB-IV	SARMS	SARMS	NSCLC	121
Douillard et al. [24]	Caucasian	Prospective	Plasma	NA	III, IV	SARMS	SARMS	NSCLC	652
Zhu et al. [25]	Asian	NA	Plasma	NA	IIIB-IV	ARMS	ddPCR	ADC, ADSQC	86
Mok et al. [26]	Asian	Prospective	NA	2	IIIB-IV	allele-specific PCR	allele-specific PCR	NSCLC	238
Rachiglio et al. [27]	Caucasian	NA	Plasma	10	III-IV	NGS	NGS	NSCLC	44
Ma et al. [28]	Asian	NA	Plasma	20	III-IV	ARMS	ARMS	NSCLC	219
Chai et al. [29]	Asian	NA	Plasma	10	III, IV	ARMS	cSMART	ADC, SQCC	61

NA: not mention; ARMS: amplification refractory mutation system; DHPLC: denaturing high performance liquid chromatography; ddPCR: droplet digital PCR; ME-PCR: mutant-enriched polymerase chain reaction; SARMS: scorpions amplification refractory mutation system; NGS: next-generation sequencing; cSMART: circulating single-molecule amplification and re-sequencing technology; ADC: adenocarcinoma; SQCC: squamous carcinoma; LCC: large cell carcinoma; NSCLC: non-small cell lung cancer.

**Figure 2:**
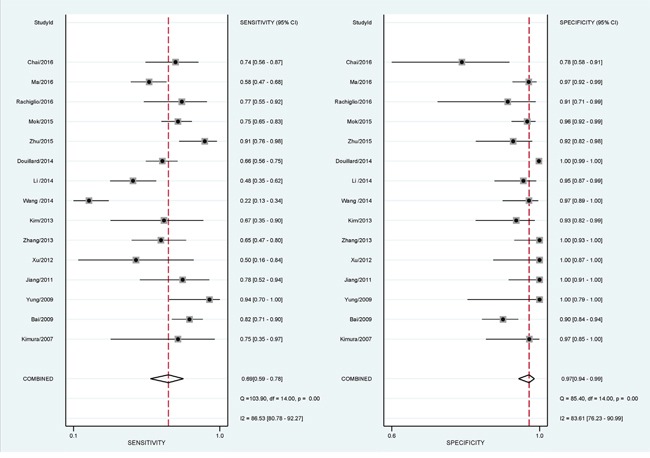
Forest plots of pooled sensitivity and specificity The plots showed the pooled sensitivity was 0.66 (95% confidence interval (CI): 0.54–0.77), and the specificity was 0.97 (95% CI: 0.94–0.99) of liquid biopsy in advanced non-small cell lung cancer patients.

**Figure 3:**
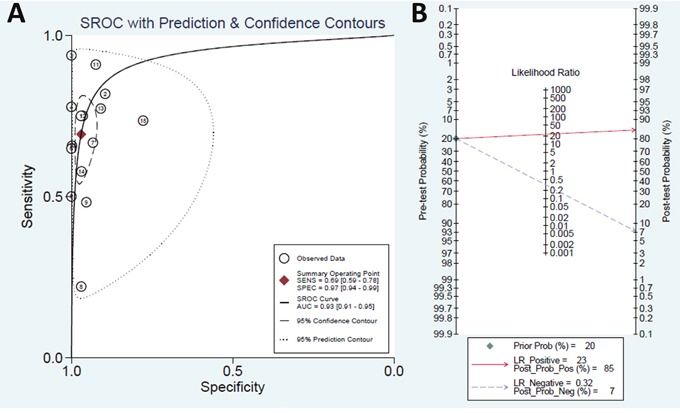
**(A)** Summary receiver operating characteristics (SROC) plot. It showed a good performance of liquid biopsy in advanced non-small cell lung cancer patients. **(B)** Fagan plot of the positive likelihood ratio and negative likelihood ratio. The plot showed a good positive likelihood ratio and a moderate negative likelihood ratio.

QUADAS-2 summary plot was presented in Supplementary Figure 1. The pooled sensitivity was 0.69 (95% confidence interval (CI): 0.59–0.78), and specificity was 0.97 (95% CI: 0.94–0.99). The results showed a positive likelihood ratio (PLR) of 23.1 (95% CI: 11.6–46.1) and a negative likelihood ratio (NLR) of 0.32 (95% CI: 0.23–0.44). The diagnostic odds ratio (DOR) was 73 (95%CI: 33–159), and the area under the curve (AUC) was 0.93 (95%CI: 0.91–0.95), showing that EGFR mutation detection in peripheral blood had a high diagnostic performance. The detailed sensitivity and specificity with 95% CI for each study are presented in a Forest plot. (Figure 2, Figure 3)

### Heterogeneity assessment and meta-regression analysis

The P value for the Spearman correlation coefficient was 0.09, confirming that the threshold-effect was not significant. The meta-regression analysis showed that the detection method applied in two specimens is the most important source of heterogeneity (P <0.05). (Table 2)

**Table 2:** Subgroup analysis

Subgroup	No. of studies	Summary sensitivity (95% CI)	p	Summary specificity (95% CI)	p
Ethnicity			0.50		0.17
Asia	13	0.69 [0.58-0.79]		0.96 [0.94-0.99]	
Non-Asia	2	0.73 [0.49-0.97]		0.99 [0.97-1.00]	
Design			0.33		0.31
Prospective	6	0.69 [0.53-0.84]		0.97 [0.95-1.00]	
Retrospective or NA	9	0.70 [0.57-0.83]		0.97 [0.93-1.00]	
Sample			0.08		0.76
Plasma	12	0.66 [0.55-0.77]		0.97 [0.95-0.99]	
Serum or NA	3	0.79 [0.63-0.94]		0.96 [0.91-1.00]	
Same detection Methods			0.02		0.93
No	4	0.81 [0.68-0.93]		0.90 [0.83-0.97]	
Yes	11	0.64 [0.53-0.75]		0.98 [0.97-0.99]	
Blood			0.51		0.54
>6ml	3	0.70 [0.49-0.91]		0.92 [0.82-1.00]	
≤6ml or NA	12	0.69 [0.58-0.80]		0.98 [0.96-0.99]	

### Subgroup analysis

The subgroup with same method applied in both blood and tumor tissue had a poor pooled sensitivity (0.64, 95% CI: 0.53–0.75) than the subgroup with different methods applied in two specimen (0.81, 95% CI: 0.68–0.93)(P = 0.02). No statistically significant differences were found between the pooled specificities of the studies with same method applied in both specimen (0.98, 95% CI: 0.97–0.99) and that of the studies with different method applied in both specimen (0.90, 95% CI: 0.83–0.97) (P = 0.93).

**Figure 4:**
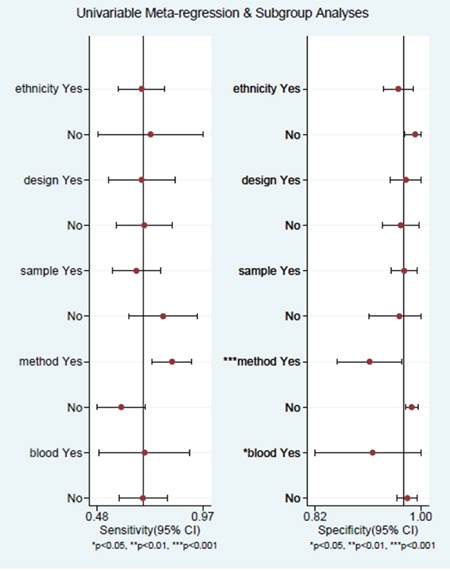
Forest plots of subgroup: analyses for sensitivity and specificity No significant different were found between the pooled specificities of the studies with ethnicity, design, sample, blood, method. A significant different were found between the pooled sensitivities of the studies with method (same methods in specimens versus different methods in specimens). The other subgroups (ethnicity, design, sample) didn't showed a significant different of sensitivity.

No significant difference was found between the pooled sensitivities and specificities of the studies with ethnicity (Asian versus non-Asian), design (prospective versus retrospective or unspecified), sample type (plasma versus serum or mixed), blood amount (>6ml versus <=6ml or unspecified). The results of the subgroup analysis are shown in Table 2 and Figure 4.

### Publication bias

As shown in Supplementary Figure 2, the P value of 0.28 (P>0.05) suggested no publication bias observed.

## DISCUSSION

The development of EGFR-TKIs has revolutionized the treatment of NSCLC, benefiting a sub-population of patients harboring EGFR mutations with a median PFS of 12 months. Therefore, obtaining EGFR mutation status prior to commencing EGFR-TKIs is necessary [11]. Although tumor tissue obtained from surgery or biopsy is still regarded as the gold standard; however, it are unwilling to undergo repeat tissue biopsy. Peripheral blood, one of the non-invasive surrogates for driver gene examination, has attracted attention. An increasing number of studies focused on liquid biopsy in NSCLC patients had published in the last few years. However, obtaining EGFR status using peripheral blood for EGFR mutation detection in aNSCLC patients is still far from clear.

In our study, the results revealed that EGFR mutation detection in peripheral blood has high specificity (0.97) and moderate sensitivity (0.69). Furthermore, the overall test performance assessed by the AUC was excellent (area under ROC = 0.93). The results also showed a good overall PLR and NLR values of 23.1 and 0.32, respectively. The results indicated that if EGFR mutations were detected in the blood of aNSCLC patients, most likely they harbor EGFR mutations in tumor burden.

In our study, we have pooled 15 published studies, including a total of 2094 aNSCLC patients. The AUC of SROC was 0.93 (95% CI: 0.91–0.95), showed an excellent overall diagnostic yield. In our meta-analysis, the mean DOR of 73 suggested that peripheral blood was reliable in detecting EGFR mutation status in aNSCLC patients. Likelihood ratios are more clinically meaningful than sensitivity or specificity values [30]. In this study, the value of PLR was 23.1, which is high enough for clinical purposes. However, the NLR value was 0.32, which is not low enough to exclude the EGFR mutation when EGFR detection in blood is negative.

It is important to note that significant heterogeneity exited among the studies analyzed in our work. But there was no threshold effect observed (p=0.09). Furthermore, we conducted meta-regression analysis to explore the source of heterogeneity. We found that the detection method applied in two specimens is responsible for the heterogeneity. In the subgroup analysis, studies with same detection method applied in two specimens had a poor sensitivity than studies with different detection methods applied. The other subgroups, including race, design, sample types or blood amount don't show significantly different sensitivities or specificities.

The level of ctDNA in healthy controls is low, but it is significantly increased in NSCLC patients, especially those patients with advanced disease [12]. Although the source of ctDNA is still not clear, many studies indicated that necrosis or apoptosis of tumor cells were the main source of the DNA fragment found in peripheral blood of cancer patients. In addition, the lysis of circulating tumor cells (CTCs) and the secretion of DNA fragment by the tumor cells also result in the increased level of ctDNA [31–33]. Jose et.al reported their successful attempt in detecting KRAS mutation in NSCLC patients with a good diagnostic yield of 0.74 [34]. Kimura et.al first reported their study of detecting EGFR mutation in serum with direct sequencing technique and obtained a moderate diagnostic yield of 0.55 [35]. However, the diagnostic yield increased to 0.73 if the scorpions amplification refractory mutation system (sARMS) technique was applied to the same cohort [36]. Interestingly, some studies also indicated that the sensitivity was associated with tumor differentiation [37].

Numerous methods were applied in liquid biopsy including mutant-enriched PCR (ME-PCR), denaturing high-performance liquid chromatography (DHPLC), high resolution melting (HRM), droplet digital PCR (ddPCR), next-generation sequencing, etc., but the sensitivity and diagnostic yield varied, partially attributing to the differential detection limit of each method. Method with low sensitivity would fail to detect mutations with low allelic frequency [38]. In addition, as the different inclusion criteria were adopted, some studies suggested that the sensitivity of liquid biopsy in early-stage patients is lower than those with aNSCLC [37, 39]. In Song's study, they even failed to detect EGFR mutations in patients with early-stage NSCLC utilizing direct sequencing technique [40]. However, several meta-analysis demonstrated that the stage of enrolled patients or the methods applied in liquid biopsy didn't influence the sensitivity and specificity [41–43]. In our study, we found that the pooled sensitivity is poor in the group utilized the same detection method.

In Wu, Luo and Mao's studies, they included the studies with different stages. In our study, only studies with aNSCLC were selected [41–43]. For most patients with early-stage NSCLC, they can benefit from surgery and the tumor tissue is adequate for genetic profiling. Advanced NSCLC patients are not candidates for surgery. They often undergo target therapy if they harbor sensitized driver gene mutations. However, about one out of three patients with aNSCLC would fail to undergo driver gene testing even in well-designed clinical trials [44–45]. Although fewer studies were selected in our study, the results were more clinically relevant than the previous three studies.

There are some limitations associated with this study, which may influence the interpretation of the results of this meta-analysis. This review may suffer from a verification bias. In some studies, they applied plasma for liquid biopsy, but in other studies, serum was used, even in one study, mixed specimen of plasma and serum was used. Several studies suggested that plasma would have a better performance than serum [23, 46]. But, in our study, no significant difference was found among the studies used plasma and the studies used serum or mixed specimen in the subgroup analysis. In the subgroup analysis, we were unable to find a difference in the diagnostic yield of the prospective studies and those retrospective studies or studies without specified type. Although no publication bias was observed, studies with positive or significant results are more likely to be published; therefore, a certain degree of publication bias must be expected.

In summary, the results of our study revealed that the detection of EGFR mutations in the peripheral blood of aNSCLC patients is a non-invasive and reliable method. Since a high specificity and moderate sensitivity were observed in this study, it is likely to detect EGFR mutations in peripheral blood as a promising surrogate instead of traditional tissue biopsies. However, more well-designed studies are required to identify patients who can benefit from liquid biopsy the most.

## MATERIALS AND METHODS

### Literature search

We carried out a computerized search of published research studies in the database of Medline, Embase, Web of Science databases and Cochrane library by using the following key words: “peripheral blood OR plasma OR serum” AND“epidermal growth factor receptor” AND “mutation” AND “non-small cell lung cancer”. Alternative spellings and abbreviations were also considered. Reference lists of included studies and relevant reviews were also manually searched. The literature search was conducted without any limitations. Literature published prior to Oct 2016 in English language were considered. Conference abstracts or letters to journal editors were excluded because of their limited data.

### Inclusion criteria

All potentially relevant studies met the following criteria were retrieved and assessed for inclusion: (1) the study must compare EGFR mutation statuses of peripheral blood and tumor tissues; (2) the study should include sufficient data (true-positives, false-positives, true-negatives, and false-negatives) for the calculation of following parameters: sensitivity, specificity, PLR, NLR and DOR; (3) no going treatment (including chemotherapy, radiotherapy, target therapy, etc) between blood collection and tissue collection; (4) all patients included should diagnose with aNSCLC, ranging from IIIA to~IV according to the latest TNM stage of lung cancer [47]. If the same study cohort was appeared in several articles, only the latest article was selected. If several methods were applied in one cohort, only the best result was selected. Disagreements were resolved by discussion.

### Data extraction

Data were extracted from all selected studies by two reviewers working independently, using a standardized form to ensure that all relevant information was captured. The following data were extracted from each publication: author, publication year, ethnicity, study design, peripheral blood specimen type (plasma or serum), the amount of peripheral blood required, tumor stage of the patients enrolled, pathological type, method used for EGFR mutation detection in peripheral blood specimen, method used for EGFR mutation detection in tumor tissue, total number of patients enrolled, the distribution of true-positives, false-positives, true-negatives and false-negatives. Any missing data were treated as “not reported”. No minimum number of patients for a study was required to be included in our meta-analysis. Two reviewers assessed the trial methodology of diagnostic studies according to the QUADAS scoring system [48]. The third author assessed the data and resolved the disagreement.

### Statistical analysis

All analysis was conducted according to the standard methods recommended for a meta-analysis of diagnostic test evaluations [49]. All calculations were carried out with the STATA version 12.0 statistical software package (Stata Corp., College Station, TX, USA) with the “midas” program. For each study, we calculated the following five parameters: sensitivity, specificity, PLR, NLR and DOR. All the analysis was based on a summary receiver-operator characteristic (SROC) curve [49–50]. The bivariate regression model was used to calculate the pooled sensitivity, specificity and the other parameters [51]. The likeslihood χ^2^ test and I^2^ statistics were used to detect statistically significant heterogeneity across the studies. An I^2^ value over 50% was an index of lack of heterogeneity between studies. For the likelihood ratio χ^2^ test, apparent heterogeneity existed if P < 0.05. When heterogeneity was detected, the Spearman correlation coefficient would calculate to judge whether the threshold effect existed or not. Then meta-regression and subgroup was done to explore other sources of between-study heterogeneity. Covariates included ethnicity (Asian or non-Asian), the study design (prospective or not), sample (plasma or not), the amount of peripheral blood (if more than 6ml), method applied in peripheral blood and tumor tissue (same or not). The potential publication bias was estimated by Deeks’ funnel plots [52].It was considered a statistically significant publication bias existed if the P value is less than 0.1.

## SUPPLEMENTARY MATERIALS AND FIGURES


