# Supplementary material for: Can peripheral blood be used as surrogate in detecting epidermal growth factor receptor mutation status in advanced non-small cell lung cancer patients? A meta-analysis
Source: Oncotarget. 2017 Aug 16;8(44):78057–67. doi: 10.18632/oncotarget.20291 (PMC5652836; doi:10.18632/oncotarget.20291)
Supplement: Supplementary file 1 [file oncotarget-08-78057-s001.pdf]

Can peripheral blood be used as surrogate in detecting epidermal growth factor receptor mutation status in advanced non-small cell lung cancer patients? A meta-analysis

SUPPLEMENTARY MATERIALS

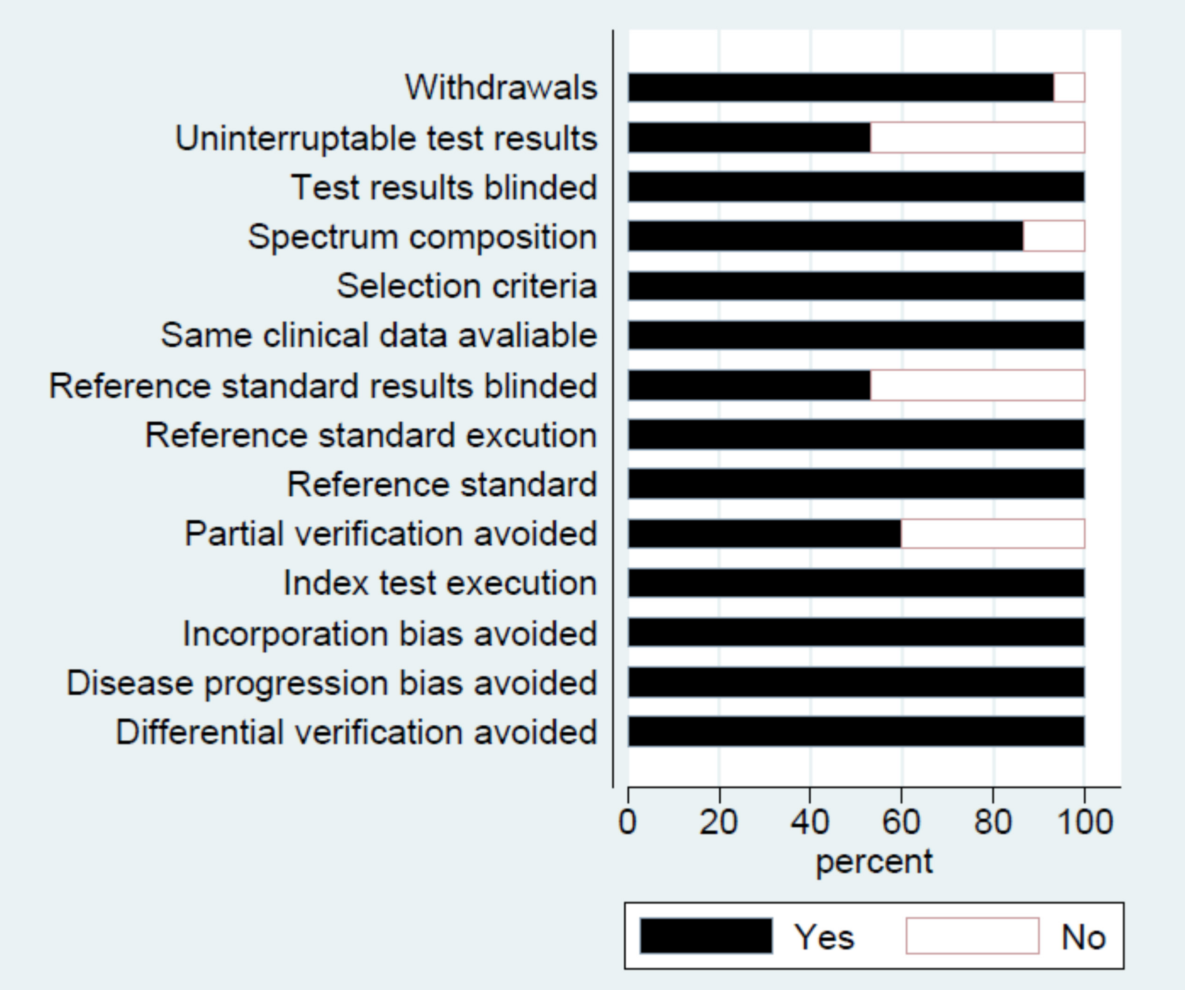

Supplementary Figure 1: Quality assessment of included studies.

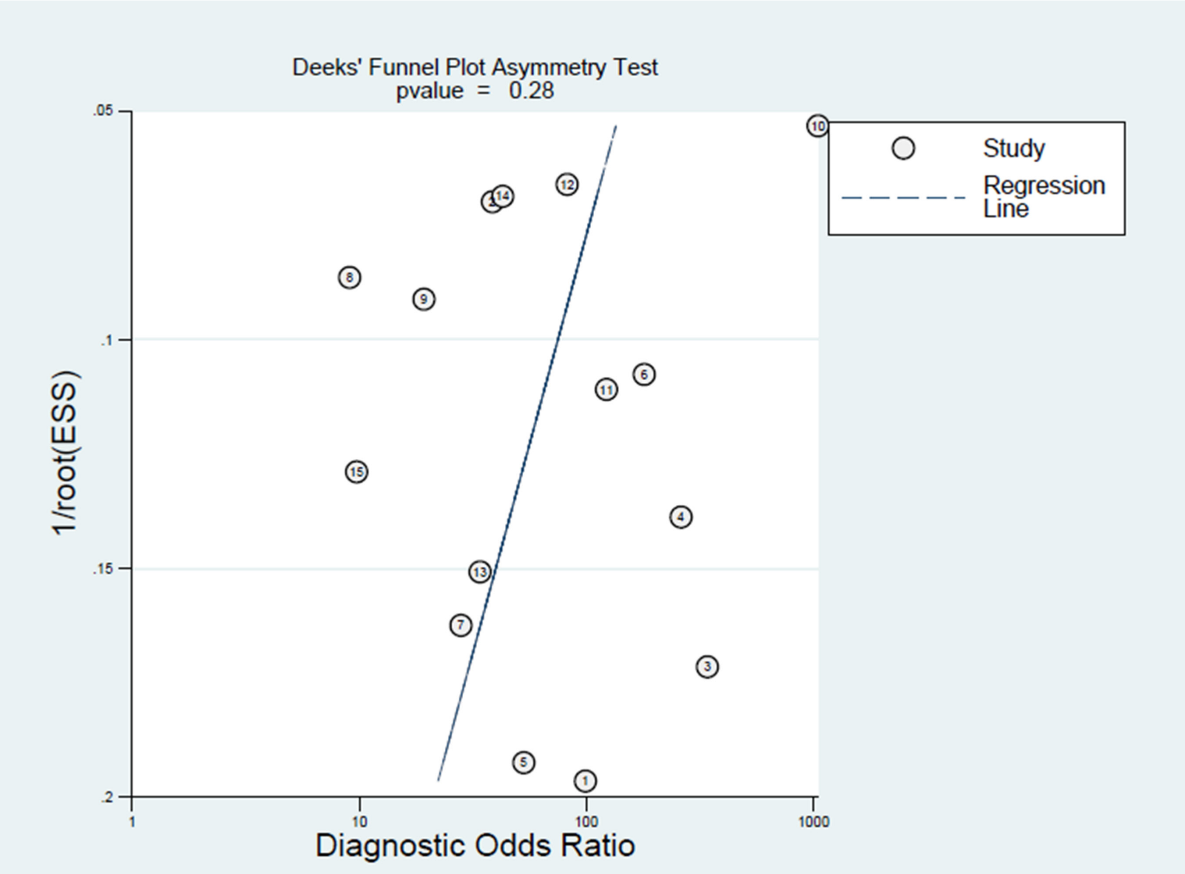

Supplementary Figure 2: Deek's funnel plot showed no significant publication bias.
